# Cigarette smoke-promoted acquisition of bacterial pathogens in the upper respiratory tract leads to enhanced inflammation in mice

**DOI:** 10.1186/s12931-015-0204-8

**Published:** 2015-03-20

**Authors:** Meike Voss, Bodo Wonnenberg, Anja Honecker, Andreas Kamyschnikow, Christian Herr, Markus Bischoff, Thomas Tschernig, Robert Bals, Christoph Beisswenger

**Affiliations:** Department of Internal Medicine V – Pulmonology, Allergology and Respiratory Critical Care Medicine, Saarland University, 66421 Homburg/Saar, Germany; Institute of Medical Microbiology and Hygiene, Saarland University, 66421 Homburg/Saar, Germany; Institute of Anatomy and Cell Biology, Saarland University, 66421 Homburg/Saar, Germany

**Keywords:** Cigarette smoke, Bacterial colonization, Inflammation, COPD

## Abstract

**Background:**

Bacterial colonization and recurrent infections of the respiratory tract contribute to the progression of chronic obstructive pulmonary disease (COPD). There is evidence that exacerbations of COPD are provoked by new bacterial strains acquired from the environment. Using a murine model of colonization, we examined whether chronic exposure to cigarette smoke (CS) promotes nasopharyngeal colonization with typical lung pathogens and whether colonization is linked to inflammation in the respiratory tract.

**Methods:**

C57BL/6 N mice were chronically exposed to CS. The upper airways of mice were colonized with nontypeable *Haemophilus influenzae* (NTHi) or *Streptococcus pneumoniae*. Bacterial colonization was determined in the upper respiratory tract and lung tissue. Inflammatory cells and cytokines were determined in lavage fluids. RT-PCR was performed for inflammatory mediators.

**Results:**

Chronic CS exposure resulted in significantly increased numbers of viable NTHi in the upper airways, whereas NTHi only marginally colonized air-exposed mice. Colonization with *S. pneumoniae* was enhanced in the upper respiratory tract of CS-exposed mice and was accompanied by increased translocation of *S. pneumoniae* into the lung. Bacterial colonization levels were associated with increased concentrations of inflammatory mediators and the number of immune cells in lavage fluids of the upper respiratory tract and the lung. Phagocytosis activity was reduced in whole blood granulocytes and monocytes of CS-exposed mice.

**Conclusions:**

These findings demonstrate that exposure to CS impacts the ability of the host to control bacterial colonization of the upper airways, resulting in enhanced inflammation and susceptibility of the host to pathogens migrating into the lung.

## Introduction

Respiratory diseases associated with cigarette smoke (CS) are a leading cause of morbidity and mortality worldwide. The most prominent respiratory disease caused by CS is chronic obstructive pulmonary disease (COPD) and it is most likely that it will constitute the fourth most common cause of death by the year 2030 [1]. A hallmark of COPD is chronic inflammation of the lung, leading to tissue destruction. The ongoing tissue destruction leads to emphysema and loss of pulmonary function [2,3].

Stable COPD patients are frequently colonized by bacterial pathogens, such as nontypeable *H. influaenzae*, *Pseudomonas aeruginosa*, and *S. pneumoniae* [2,4,5]. Moreover, recurrent infections of the respiratory tract and chronic bronchitis are common in COPD [2-5]. It is suggested that acute bacterial lung infections as well as chronic bacterial colonization of the lung contribute to the progression of COPD by amplifying inflammation which further promotes lung damage and impacts innate lung defence [2]. In addition, pathogenic bacteria cause direct damage to the epithelial surfaces of the lung and thereby contribute to impaired mucociliary clearance, elevated mucus secretion and subsequent mucus retention, disrupted ciliary activity, and finally airflow obstruction [3,6,7].

There is evidence that exacerbations are provoked by the acquisition of new bacterial strains from the environment by the host [2,3]. It is hypothesized that newly-acquired bacterial pathogens trigger infections of the lung and evoke harmful inflammatory responses, contributing to clinical deterioration of the patient during exacerbation [2,3,8]. Among the most frequent bacterial pathogens isolated from patients suffering from exacerbation are *H. influenzae* (20–30% of patients) and *S. pneumoniae* (10–15% of patients) [2].

Several studies have shown that CS has an impact on the immune system of the respiratory tract, even in healthy individuals [9,10]. Thus, smoking and passive smoking are associated with disordered microbial communities of the upper airways, impairment of commensal airway flora, and an increased risk of bacterial infections in healthy individuals [4,11-14]. Brook and Goober demonstrated that the nasopharyngeal flora of smokers contains fewer aerobic and anaerobic organisms with interfering capabilities and more potential pathogens, such as *H. influenzae* and *S. pneumoniae*, compared with those of non-smokers [12]. It is also suggested that smoking enhances colonization of the oral cavity with periodontal pathogens, affects the sub-gingival microflora in periodontitis, and is a risk factor for periodontal disease [15,16]. Moreover, cigarette smoking increases the risk of community-acquired pneumonia [17,18] and is a strong independent risk factor for invasive pneumococcal disease among immunocompetent, non-elderly adults [19]. Exposure to passive smoke favors bacterial colonization and infection in infants [12-14,20]. Greenberg *et al.* reported that children of smoking parents have a significantly higher rate of *S. pneumoniae* carriage than children of non-smokers [14]. Exposure to passive smoke also correlates with a significantly increased risk of infants developing infections of the lower respiratory tract in the first two years of life [20].

It was the aim of the present study to investigate whether chronic exposure to CS promotes nasopharyngeal colonization with the leading gram-positive and gram-negative bacterial lung pathogens *H. influenzae* and *S. pneumoniae* by combining a clinically relevant murine model of colonization of the upper respiratory tract with a model of chronic CS exposure [21]. It was further investigated whether CS-promoted colonization of the upper-respiratory tract results in increased inflammation and bacterial translocation into the lung.

## Methods

### Bacterial strains and products

A patient isolate of *H. influenzae* (ATCC® 49247™) was grown overnight at 37°C and 5% CO_2_ on chocolate agar (Becton Dickinson, Germany). Bacteria were taken from the plate and re-suspended in 7 ml 1 × PBS to obtain an optical density of 1 at 600 nm. Bacteria were spun at 4000 rpm and 4°C for 10 minutes and re-suspended in 350 μl 1 × PBS for the infection. A type 6A clinical isolate of *S. pneumoniae* (a gift from Dr. Jeffrey Weiser, University of Pennsylvania) [22,23] was cultured in Todd Hewitt broth at 37°C. Mid-log-phase *S. pneumoniae* were washed with PBS and re-suspenended in 450 μl 1 × PBS.

### CS exposure and nasopharyngeal colonization

All animal experiments were approved by the Landesamt für Soziales, Gesundheit und Verbraucherschutz of the State of Saarland following the national guidelines for animal treatment. Mice were maintained under a pathogen-free condition. 7 to 9 week-old female wild-type C57BL/6 N mice were exposed to a combination of sidestream and mainstream CS (3R4F, College of Agriculture, Reference Cigarette Program, University of Kentucky, Lexington, Kentucky, USA) in a TE-10 smoking machine (Teague Enterprises, Woodland, California, USA) for a total of 261 minutes/day, 5 days per week. The smoking time was 87 minutes with 40 minutes air exposure in between CS exposures, with 3 CS exposures/day. Mice were exposed to CS or air for 13 (3 months) or 30 weeks (7 months) as indicated in the figures. The CS concentration was 120 mg/m^3^ total suspended particles. 24 hours after the final CS exposure the upper airways of room air- and CS-exposed mice were colonized using an established model of nasopharyngeal colonization [22-24]. Mice were inoculated intranasally with 10 μl of mid-log phase NTHi (5.5 × 10^6^ to 4.5 × 10^7^ CFU) or *S. pneumoniae* 6A (1.5 × 10^7^ to 2.5 × 10^7^ CFU) applied atraumatically to the nares without anesthesia.

### Lavage of the upper airways and bronchoalveolar lavage and phagocytosis

Lavage of the upper airways and bronchoalveolar lavage (BAL) were performed as described before [22,23]. Briefly, the animals were sacrificed and the trachea were cannulated. Lavage of the upper airways was performed with 300 μl of PBS. To obtain BAL fluids from the lung BAL was performed with 1 ml of PBS flushed three times into the lungs. Lavage fluids were plated in serial dilutions and CFUs were determined. Lavage fluids were centrifuged to obtain immune cells and supernatants. Cell numbers were determined. Inflammatory cells were differentiated on cytospins. Serum was obtained by cardiac puncture. Phagocytosis activity of whole blood monocytes and granulocytes obtained from mice exposed to air or CS for 3 months was determined using a flow cytometry-based assays (Phagotest, Orpegen Pharma, Germany), according to the instruction. Samples were analyzed in a FACS Calibur flow cytometer (Beckton Dickinson, Heidelberg, Germany). Gates (forward/side scatter) were set on granulocytes and monocytes. For determination of the phagocytosis activity in each sample the mean fluorescence intensity was calculated according to the instruction of the manufacturer (MFI).

### Determination of cytokine concentrations and immunohistochemistry

Concentrations of inflammatory mediators were assessed by cytometric bead array (CBA) using a FACSCanto II (BD Bioscience, Germany). For immunohistochemistry of the upper respiratory tract, the heads of the mice were fixed in 4%-PBS-buffered formaldehyde and decalcified [22]. Immunohistochemistry on paraffin sections was performed as described before, using anti-*H. influenzae* (abcam, USA) antibody [25,26].

### Quantitative real-time PCR

Total RNA from the upper airways was isolated after flushing the upper airways to obtain cells [23]. The upper airways were flushed with 300 μl RLT Lysis buffer (Qiagen, Germany). RNA was isolated and reverse transcription and real-time PCR were performed as described before [27]. Primers were: mouse β-Actin: 5′-AGC CAT GTA CGT AGC CAT CC-3′ and 5′-CTC TCA GCT GTG GTG GTG AA-3′, mouse Lysozyme M: 5′-CTG GCT ACT ATG GAG TCA GC-3′and 5′-TTG ATC CCA CAG GCA TTC AC-3′, mouse MIP-2: 5′-AAG TTT GCC TTG ACC CTG AA-3′ and 5′-AGG CAC ATC AGG TAC GAT CC-3′, and mouse mBD-1: 5′-GGC TGC CAC CAC TAT GAA AAC TC-3′ and 5′-GAG ACA GAA TCC TCC ATG TTG AA-3′.

### Statistical analysis

Values are displayed as mean ± SEM. The Mann–Whitney test was used to compare the groups. Results were considered statistically significant for p < 0.05. All statistical tests were performed using the software Prism (GraphPad Software, San Diego, CA, USA).

## Results

### CS exposure promotes colonization of the upper airways with NTHi and *S. pneumoniae*

**Figure 1:**
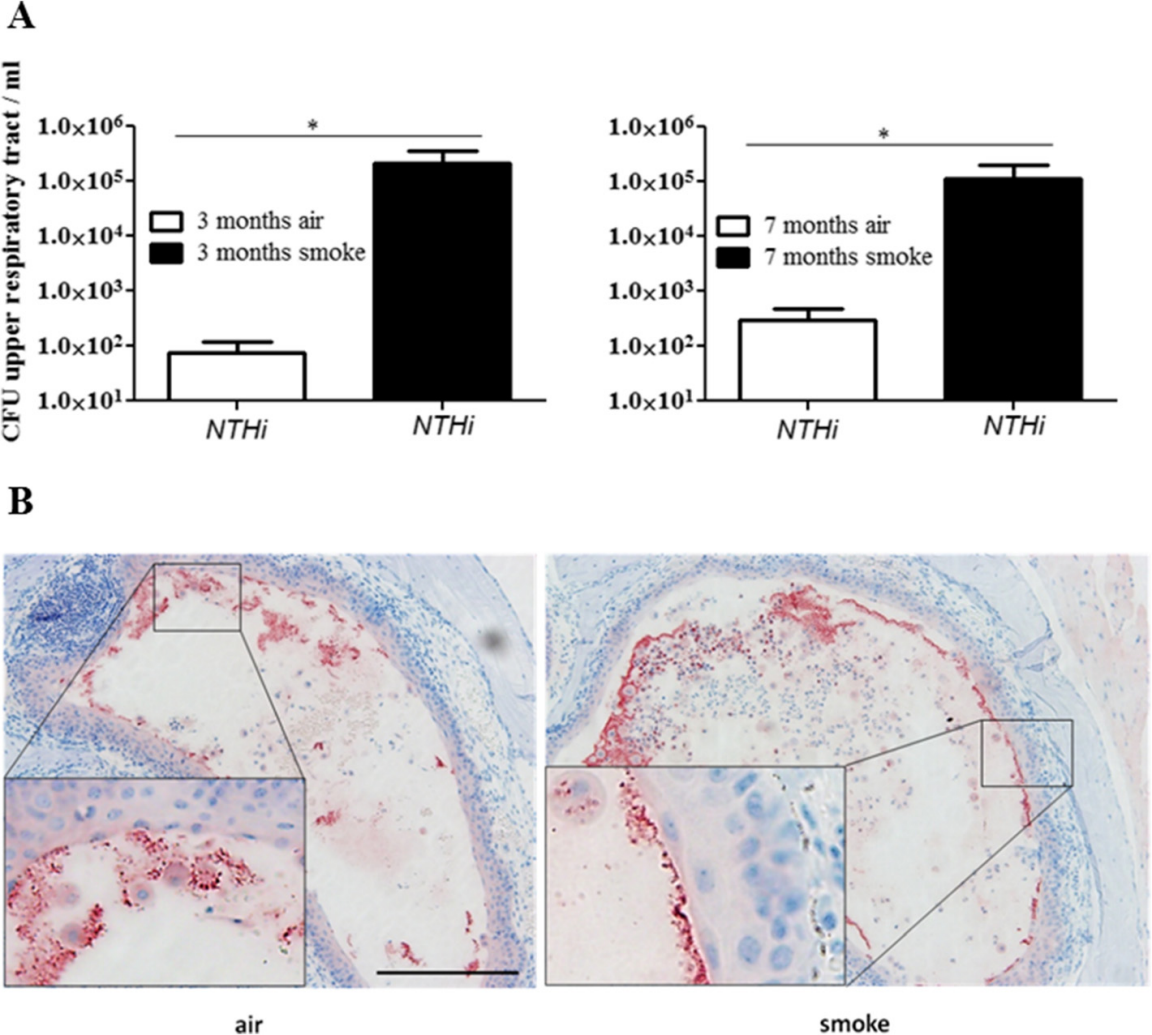
**Colonization levels in the upper airways of mice inoculated with NTHi. (A)** Mice were exposed to CS or air as indicated and intranasally inoculated with NTHi. Colonization densities were determined by quantitative cultures of upper airway lavages 24 hours postinoculation. Data are shown as mean ± SEM. Bars indicate significant differences of *p < 0.05, n ≥ 5 per group. **(B)** NTHi are shown in nasal tissue by immunohistochemistry (red staining). Scale bar, 100 μm.

**Figure 2:**
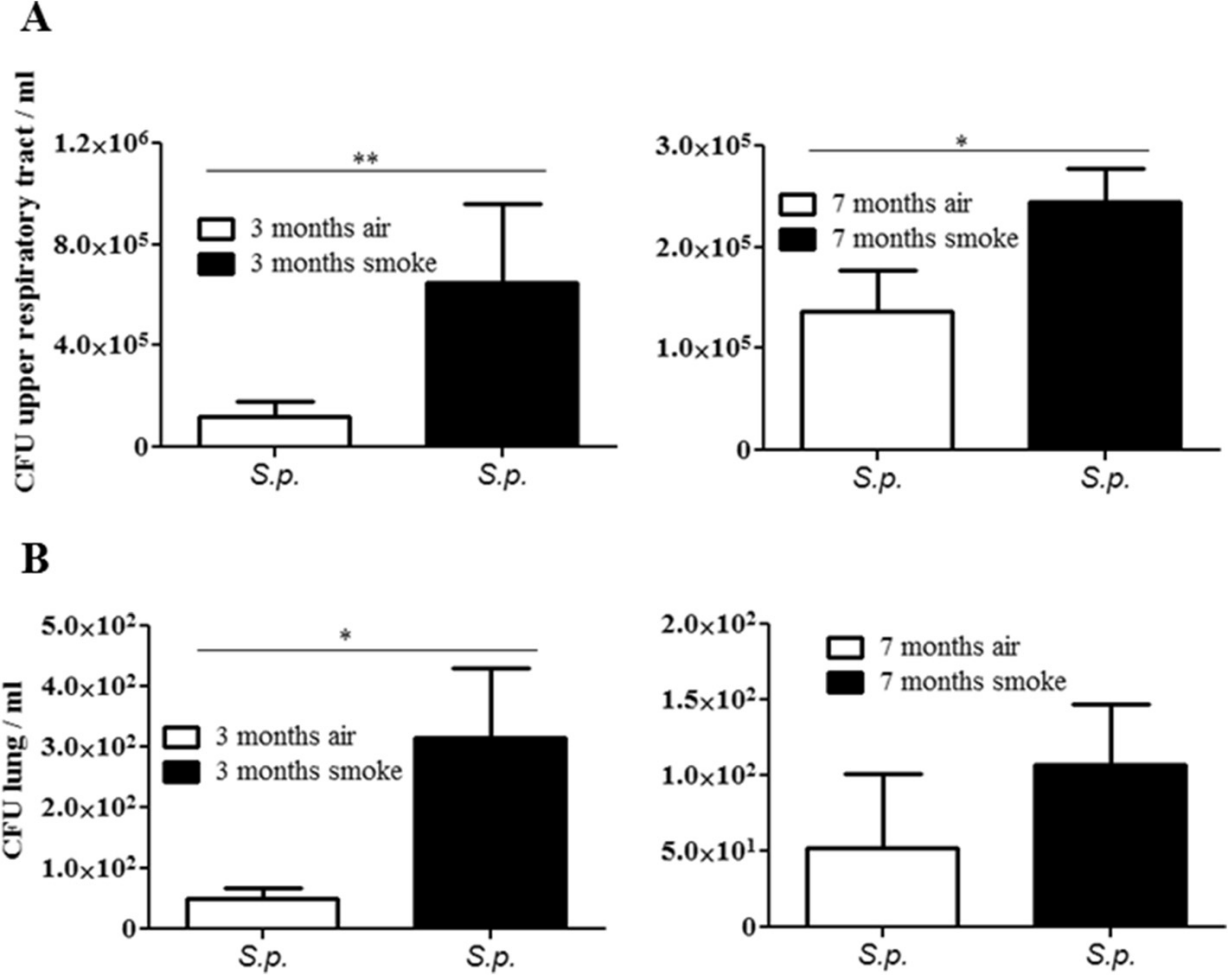
**Colonization levels in the upper airways of mice inoculated with** ***S. pneumoniae*** **. (A)** Mice were exposed to CS or air as indicated and intranasally inoculated with *S. pneumoniae* (*S.p.*). Colonization densities were determined by quantitative cultures of upper airway lavages 4 days (3 months CS) and 7 days (7 months CS) postinoculation. **(B)** Numbers of viable *S.p.* were determined in lung tissue (homogenate). Bars indicate significant differences of *p < 0.05 and **p < 0.01, n ≥ 5 per group.

To investigate whether exposure to CS enhances initial nasopharyngeal colonization, mice were chronically exposed to CS or room air for 3 or 7 months before intranasal inoculation with a nontypeable patient isolate of *H. influenzae* (NTHi) or an *S. pneumoniae* 6A strain, using a physiologically relevant and well-characterized murine model of colonization of the upper airways [22-24,28]. In this colonization model, stable colonization of *S. pneumoniae* occurs at the epithelial surfaces of the upper airways over period of 14 days before bacteria are cleared by cellular effectors, whereas NTHi are efficiently cleared within 24 hours [22-24,29]. Thus, we examined the effect of CS on initial nasopharyngeal colonization of *S. pneumoniae* 4 and 7 days post-inoculation and on nasopharyngeal colonization of NTHi 24 hours post-inoculation. In line with the literature, only marginal numbers of viable NTHi were determined in upper airway lavages of air-exposed mice 24 hours post-inoculation, whereas *S. pneumoniae* efficiently colonized the upper airways of air-exposed mice over a period of 7 days (Figures 1A and 2A). Chronic exposure to CS for 3, respectively 7 months, resulted in significantly increased colonization levels in the upper airways of mice inoculated with NTHi or *S. pneumoniae*, as compared to those exposed to air (Figures 1A and 2A). Colonization densities of NTHi were three magnitudes greater in the upper airways of mice exposed to CS for 3 and 7 months 24 hours after inoculation, as compared to corresponding air-exposed mice (Figure 1A). Figure 1B shows NTHi in the upper respiratory tract of room air- and CS-exposed mice on nasal sections. Moreover, in the case of inoculation with *S. pneumoniae*, colonization densities in the upper airways were 5 times higher in mice exposed to CS for 3 months and 2 times higher in mice exposed to CS for 7 months, 4, respectively 7, days after inoculation (Figure 2A).

### CS exposure leads to increased translocation of *S. pneumoniae* into the lung

We examined whether increased colonization of the upper airways correlated with enhanced bacterial numbers in the lung. To determine the number of viable bacteria in lung tissue, the lungs were dissected and the trachea was removed. CFUs were determined in lung homogenates. Viable NTHi were not detectable in the lung tissue of either CS- or air-exposed mice. Only small numbers of viable *S. pneumoniae* were determined in the lung tissue of both air- and CS-exposed mice (Figure 2B). The numbers of *S. pneumoniae* in the lung tissue were, however, enhanced in mice which had been chronically exposed to CS prior to bacterial colonization. Significantly increased numbers of viable *S. pneumoniae* were determined in the lung tissue of mice exposed to CS for 3 months, as compared to corresponding air-exposed mice 4 days after inoculation (Figure 2B).

### CS exposure increases the expression of inflammatory cytokines in the upper airways of mice colonized with NTHi

**Figure 3:**
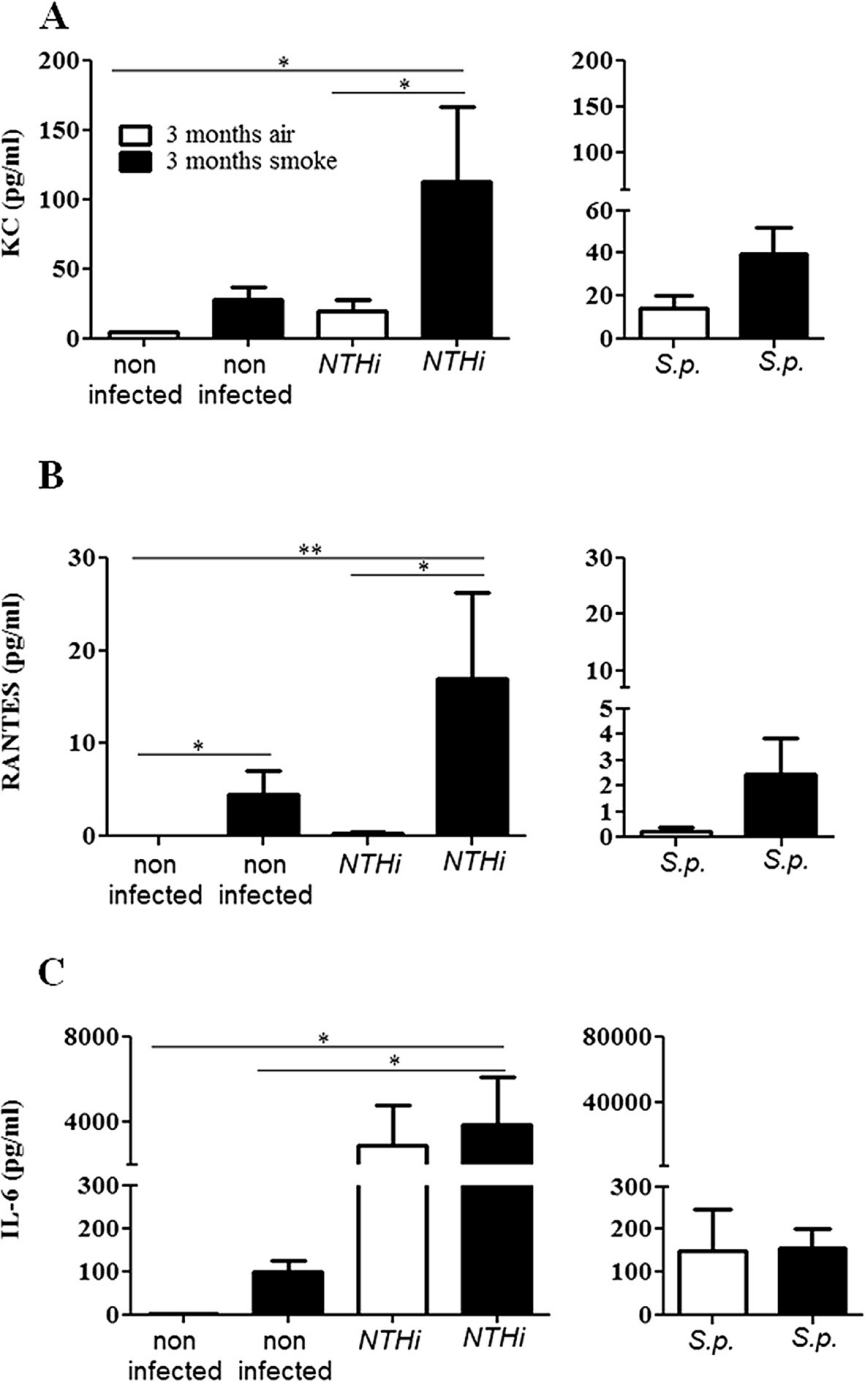
**Concentrations of inflammatory mediators in upper airway lavages of mice chronically exposed to CS and colonized with NTHi or** ***S. pneumoniae*** **.** Mice were exposed to CS or air for 3 months and intranasally inoculated with NTHi or *S. pneumoniae* (*S.p*.). Concentrations of KC **(A)**, RANTES **(B)**, and IL-6 **(C)** were measured in upper airway lavages of mice by CBA 24 hours (NTHi) and 4 days (*S.p*.) postinoculation. Data are shown as mean ± SEM. Bars indicate significant differences of *p < 0.05 and **p < 0.01, n ≥ 5 per group.

**Figure 4:**
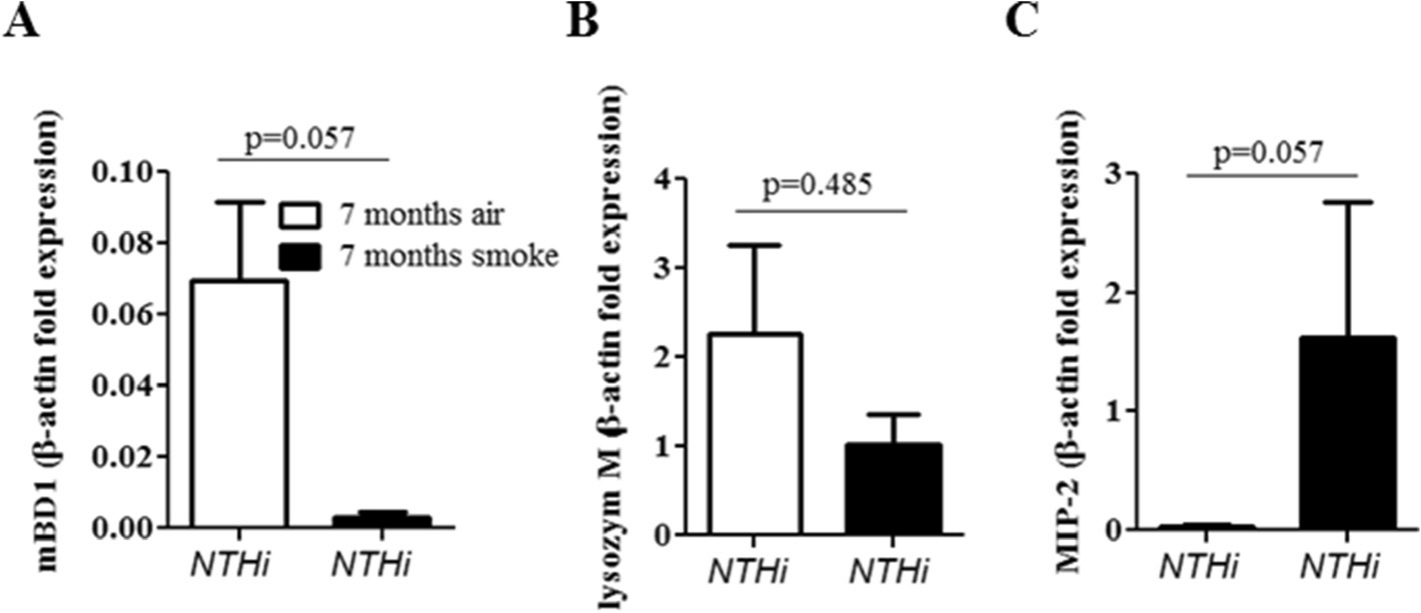
**Expression of inflammatory mediators in the upper respiratory tract.** Mice were exposed to CS or air for 7 months and intranasally inoculated with NTHi. Total RNA was obtained by flushing the upper airways with lysis buffer. The expression of mBD-1 **(A)**, lysozyme **(B)** and MIP-2 **(C)** was determined by qRT-PCR. Data are shown as mean ± SEM. n = 4 per group.

We further examined whether chronic exposure to CS exacerbates upper airway inflammation induced by colonizing bacteria. Figure 3 shows that concentrations of inflammatory mediators were increased in upper airway lavages of mice chronically exposed to CS and colonized with NTHi or *S. pneumoniae*, as compared to colonized air-exposed mice. Concentrations of KC, a major murine chemoattractant for neutrophils, were significantly increased in upper airway lavages of mice exposed to CS and colonized with NTHi, as compared to air-exposed mice colonized with NTHi and non-infected mice (Figure 3A). CS exposure and colonization with NTHi alone did not result in significantly increased concentrations of KC as compared to air-exposed control mice. Exposure to CS slightly - however not significantly (p = 0.072) - increased concentrations of KC in upper airway lavages of mice colonized with *S. pneumoniae* (Figure 3A). Similar to KC, concentrations of RANTES (also named CCL5), a major chemoattractant for monocytes, were significantly increased in upper airway lavages of mice exposed to CS and colonized with NTHi, as compared to air-exposed mice colonized with NTHi and non-infected mice (Figure 3B). However, exposure to CS alone also led to significantly increased concentrations of RANTES in upper airway lavages, as compared to non-infected control mice, whereas colonization of air-exposed mice with NTHi or *S. pneumoniae* did not lead to increased concentrations of RANTES. Colonization of air-exposed mice with NTHi or *S. pneumonaie* led to increased concentrations of IL-6 in upper airway lavages, which was not further increased in animals exposed to CS (Figure 3C). We further examined the expression of lysozyme, the antimicrobial peptide mBD-1 and the chemokine MMP-2 by qRT-PCR with RNA obtained from the upper airways of mice colonized with NTHi. It has been shown before that mBD-1 deficient mice are more susceptible to *H. influenzae* lung infection [30]. Chronic exposure to CS suppressed the expression of the antimicrobial peptide mBD-1 and increased the expression of the chemokine MIP-2 whereas the expression of lysozyme was not affected by CS (Figure 4).

### CS exposure does not inhibit the recruitment of inflammatory cells in the upper airways of colonized mice

**Figure 5:**
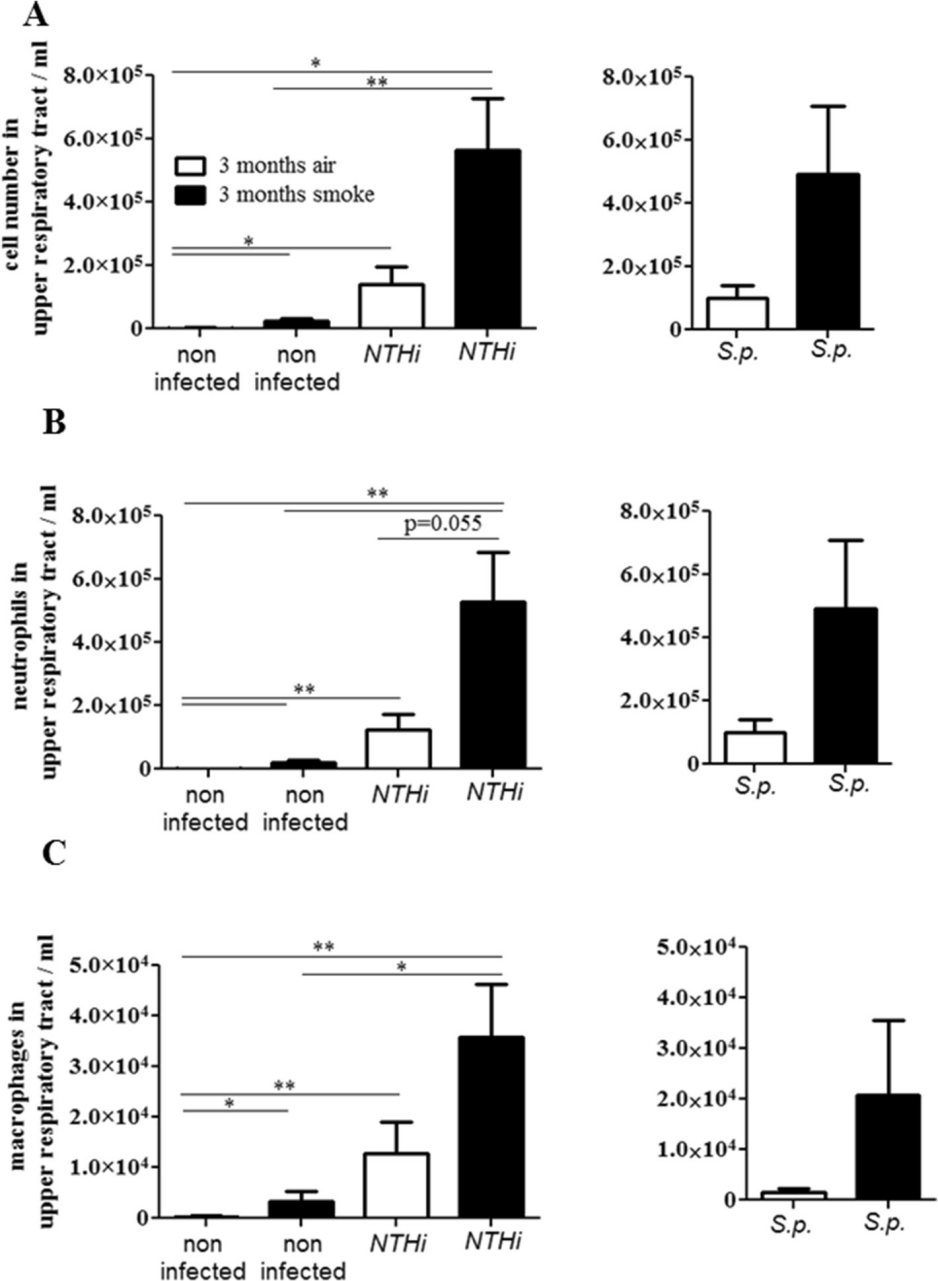
**Influx of inflammatory cells in the upper airways.** Mice were exposed to CS or air for 3 months and intranasally inoculated with NTHi or *S. pneumoniae* (*S.p*.). Numbers of total immune cells **(A)**, neutrophils **(B)** and macrophages **(C)** were determined in upper airway lavages 24 hours (NTHi) and 4 days (*S.p*.) postinoculation. Data are shown as mean ± SEM. Bars indicate significant differences of *p < 0.05 and **p < 0.01, n ≥ 5 per group.

We determined the total numbers of inflammatory cells in upper airway lavages and differentiated macrophages and neutrophils on cytospins. Exposure to CS and bacterial colonization led to a significantly increased influx of total immune cells (Figure 5A), neutrophils (Figure 5B), and macrophages (Figure 5C) into the upper airways. The number of neutrophils in upper airway lavages was approximately one magnitude higher than the number of macrophages. Numbers of total immune cells, neutrophils, and macrophages were significantly increased in upper airway lavages of mice exposed to CS and colonized with NTHi, as compared to non-infected air- and CS-exposed mice (Figure 5). The number of total immune cells, neutrophils, and macrophages was increased in upper airway lavages of CS-exposed NTHi-colonized mice compared to those of air-exposed NTHi-colonized mice. However, the differences did not reach statistical significance.

### The phagocytosis activity is reduced in whole blood granulocytes and monocytes

**Figure 6:**
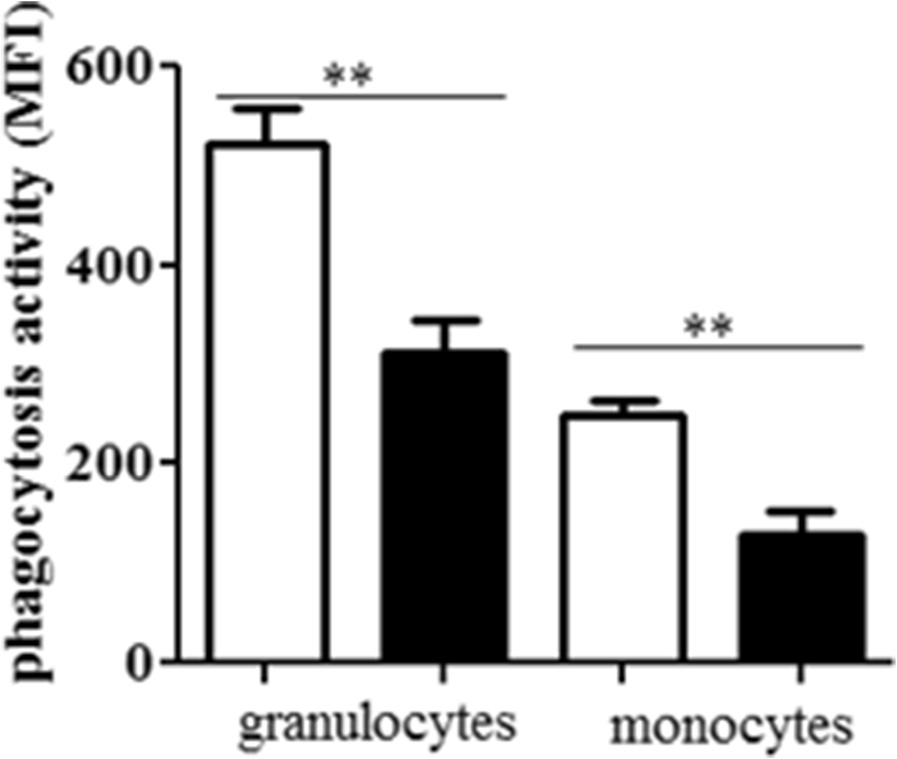
**Effect of CS on** ***E. coli*** **-induced phagocytosis.** Whole blood samples were obtained from mice exposed to CS or air for 3 months and incubated with FITC-conjugated *E. coli*. Phagocytosis activity is shown as mean fluorescence intensity (MFI). Data are shown as mean ± SEM. Bars indicate significant differences of **p < 0.01, n = 3 per group in duplicates.

Having shown that the influx of immune cells into the upper airways of colonized mice is not inhibited by CS, we determined whether the phagocytosis activity of granulocytes and monocytes was affected by exposure to CS. As there are only marginal numbers of granulocytes and monocytes present in the upper airways of non-colonized animals, we determined the ability of granulocytes and monocytes obtained from anticoagulated whole blood to phagocytose FITC-conjugated *Escherichia coli*. The phagocytosis activity was reduced in granulocytes and monocytes of mice exposed to CS for 3 months compared to those of air-exposed mice (Figure 6).

### Bacterial colonization is linked with an increased recruitment of immune cells into the lung

**Figure 7:**
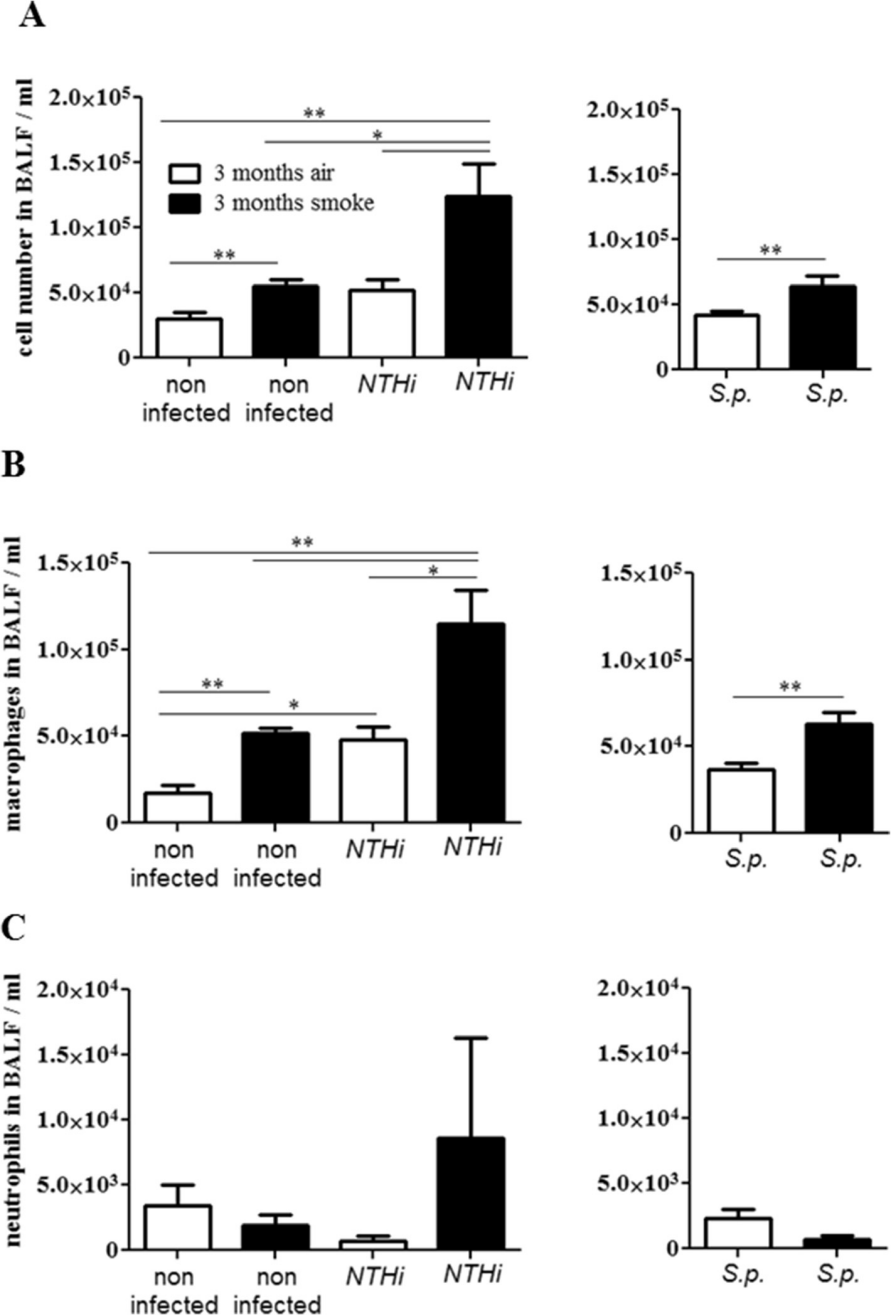
**Influx of inflammatory cells in the lung.** Mice were exposed to CS or air for 3 months and intranasally inoculated with NTHi or *S. pneumoniae* (*S.p*.). Numbers of total immune cells **(A)**, macrophages **(B)** and neutrophils **(C)** were determined in BAL fluids 24 hours (NTHi) and 4 days (*S.p*.) postinoculation. Bars indicate significant differences of *p < 0.05 and **p < 0.01, n ≥ 5 per group.

To examine whether chronic exposure to CS and bacterial colonization affects recruitment of immune cells into the lung we determined the total number of immune cells, macrophages, and neutrophils in bronchoalveolar lavage (BAL) fluids. The total number of immune cells and macrophages was significantly increased in BAL fluids of CS-exposed mice and there was an increase in macrophages in colonized air-exposed mice, as compared to air-exposed control mice (Figure 7A and B). Interestingly, there was a significant increase in the number of total immune cells and macrophages in mice chronically exposed to CS and colonized with NTHi or *S. pneumoniae,* as compared to CS-exposed control mice and colonized air-exposed mice (Figure 7A and B). In addition, we stimulated alveolar macrophages of air and CS-exposed mice ex vivo with NTHi or *S. pneumoniae* for 4 hours. There was no difference between the release of KC in alveolar macrophages obtained from CS-exposed mice and those of air-exposed mice after bacterial stimulation (data not shown).

## Discussion

We combined a murine model of colonization of the upper respiratory tract with a model of COPD to investigate whether CS-induced inflammation promotes acquisition of bacterial lung pathogens and whether colonizing of the upper airways contributes to increased inflammation in the respiratory tract. We focused on NTHi as this bacterium frequently colonizes lungs of COPD patients and is linked to exacerbations of COPD [2,5] and on *S. pneumoniae* as colonization of the nasal space by pneumococci occasionally causes pneumonia and invasive disease, especially in subjects exposed to CS or passive smoke [17-19]. Our data show that chronic exposure to CS has an impact on the ability of the host to contain bacterial colonization of the upper airways with bacterial pathogens and that CS-induced colonization is accompanied by increased inflammation of the respiratory tract and susceptibility of the host to pathogens migrating into the lung.

It is hypothesized that exacerbations in COPD are provoked by bacterial strains newly acquired from the environment [2,3]. In line with this hypothesis, our study proves that COPD-like inflammation promotes the acquisition of typical COPD pathogens, such as NTHi, into the upper airways. Increased colonization of the upper airways of CS-exposed mice with NTHi is also associated with an enhanced inflammation in the respiratory tract characterized by an increased release of chemokines and influx of immune cells into the upper airways and into the lung. Thus, our data suggest that CS promotes the acquisition and colonization of the upper airways with respiratory pathogens and that these newly- acquired pathogens contribute to the progression of COPD by enhancing and perpetuating lung inflammation [2,3,8].

For many bacterial pathogens colonization of the upper airways is often a first step in the process leading to infectious diseases [22,23]. Frequent acquisition of bacterial pathogens in the upper airways may also promote infectious lung diseases and invasive diseases in individuals exposed to mainstream or passive smoke. Our data indicate that the increased risk of smokers for community-acquired pneumonia and invasive pneumococcal disease [17-19] and the increased risk of infants exposed to passive smoke of developing infections of the lower respiratory tract [20] are a direct consequence of CS-induced acquisition of bacterial pathogens into the upper airways. Our results are further in line with findings that children of smoking parents have a significantly higher rate of *S. pneumoniae* carriage than those of non-smokers [14].

Several studies have examined how CS affects immune mechanisms at the mucosal surfaces of the respiratory tract [9,27,31-36]. In vitro studies have shown that exposure of respiratory epithelial cells to CS results in the suppression of cellular signaling cascades (e.g. AP-1- and NF-κB-dependent signaling cascades) that are of key importance in the activation of innate immune mechanisms in the case of microbial infections [31,35,36]. Thus, in vitro, CS exposure results in a reduced expression and release of inflammatory mediators and antimicrobial peptides by respiratory epithelial cells infected with bacterial pathogens [9,27,31,35,36]. Moreover, we have previously shown that smokers with community- acquired pneumonia have reduced levels of antimicrobial peptides in their sputum and pharyngeal washings [9]. In this study, we found that chronic CS exposure resulted in reduced expression levels of the antimicrobial peptide mBD-1 in the upper airways of NTHi-colonized mice, even though the colonization levels were increased in CS-exposed mice, as compared to air-exposed mice. It has been shown before that mBD-1 deficient mice are more susceptible to *H.* influenzae lung infection [30]. Furthermore, it has been shown that CS increases adhesion of bacteria to epithelial cells [37-40]. Grigg et al. showed, for instance, that CS extract stimulates platelet-activating factor receptor- dependent adhesion of *S. pneumonaie* to respiratory epithelial cells [39]. Thus, the suppression of innate immune functions of respiratory epithelial cells, including the expression of epithelial antimicrobial peptides as well as increased bacterial adherence to epithelial cells, are potential mechanisms responsible for the increased levels of colonization observed in mice chronically exposed to CS.

Studies have also shown that CS impairs the response of immune cells such as alveolar macrophages and neutrophils to bacterial pathogens, which results in a deregulated release of inflammatory mediators and impaired phagocytic activity [41-44]. Alveolar macrophages from ex-smokers with COPD, for instance, showed a reduced expression of inflammatory mediators (e.g. TNF-α) in response to antigens of *H. influaenzae,* compared to macrophages obtained from ex-smokers without COPD and non-smokers [41]. Moreover, ex vivo studies with alveolar macrophages obtained from CS-exposed mice showed that CS exposure enhances the release of the chemokines IL-1α and MCP-1 and inhibits the release of TNF-α by alveolar macrophages stimulated with NTHi [42,43]. Berenson et al. showed that the phagocytosis of NTHi is impaired in alveolar macrophages obtained from donors with COPD, as compared to that from donors without COPD [45]. Taylor et al. found that monocyte-derived macrophages from COPD patients showed reduced phagocytic responses to *H. influenza* and *S. pneumoniae,* compared with smokers and non-smokers, which was not observed by Berenson et al. [46]. Exposure to CS extract also resulted in a reduced uptake of NTHi by murine and human cell line-derived macrophages [44]. Exposure to CS extracts also reduced the complement-mediated phagocytosis of *S. pneumoniae* by alveolar macrophages ex vivo [33] and the phagocytosis of *E. coli* by human neutrophils [34]. In our model, chronic exposure to CS did not result in a defect in the recruitment of phagocytes into the upper airways in response to colonizing bacteria. Chronic CS exposure resulted in increased numbers of neutrophil and macrophages in the upper airways of mice colonized with NTHi and *S. pneumoniae*, which was associated with increased levels of chemokines in upper airway lavages. However, the phagocytosis activity of whole blood granulocytes and monocytes was inhibited in CS-exposed mice. This was a surprising and not suspected finding which might have impact on extra pulmonal research on tobacco smoke, too. Thus, it is conceivable that impaired phagocytosis of NTHi and *S. pneumoniae* by neutrophils and macrophages is another potential cause of the increased colonization levels in the upper airways of CS-exposed mice.

In summary, our data indicate that continuous exposure to CS opens a niche for typical COPD pathogens in the upper airways, which results in the acquisition of bacterial pathogens. Our data suggest that bacterial pathogens efficiently colonizing the mucosal surfaces of the upper respiratory tract contribute to increased lung colonization and are a cause of pulmonary inflammation. Frequent acquisition of bacterial pathogens into the upper airways may also promote pneumonia and invasive diseases in individuals exposed to mainstream or passive smoke. Our model system provides a tool for future mechanistic studies to examine mechanisms and potential therapeutic interventions regarding bacterial colonization and COPD exacerbations*.*
